# Supplementary material for: Long wavelength near-infrared and red light-driven consecutive photo-induced electron transfer for highly effective photoredox catalysis
Source: Nat Commun. 2024 Aug 23;15:7270. doi: 10.1038/s41467-024-50795-y (PMC11344023; doi:10.1038/s41467-024-50795-y)
Supplement: Supplementary file 1 — supplementary information [file 41467_2024_50795_MOESM1_ESM.pdf]

Supplementary Information

Long wavelength Near-infrared and Red Light-driven Consecutive Photo-induced Electron Transfer for Highly Effective Photoredox Catalysis

*Le Zeng[1,2,3,4], Ling Huang [1,3], Zhi Huang [3], Tomoyasu Mani [5], Kai Huang [1], Chunying Duan[*2] & Gang Han[*1]*

[1]Department of Biochemistry and Molecular Pharmacology, University of Massachusetts Medical School, Worcester, MA 01605, United States.

[2]State Key Laboratory of Fine Chemicals, Dalian University of Technology, Dalian, 116024, China.

[3]Tianjin Key Laboratory of Biosensing and Molecular Recognition, Research Center for Analytical Sciences, College of Chemistry, Nankai University, Tianjin 300071, P. R. China.

[4]School of Materials Science and Engineering, National Institute for Advanced Materials, TKL of Metal and Molecule-Based Material Chemistry, Nankai University, Tianjin 300350, P. R. China

[5]Department of Chemistry, University of Connecticut, 55 N. Eagleville Rd Storrs, CT 06269, United States.

Supplementary Methods

Chemicals: *N,N'*-bis(2,6-diisopropylphenyl)perylene-3,4,9,10-bis(dicarboximide) (PDI), methyl methacrylate, and ethylene glycol dimethacrylate were purchased from TCI chemicals. Meso-tetraphenyltetrabenzoporphine palladium complex (PdTPBP) was purchased from Frontier Scientific, Inc. Ethyl 2-bromopropionate was purchased from Adamas-beta, Inc. The solvents of dimethylformamide (DMF) and toluene, triethylamine (TEA) was purchased from Sigma-Aldrich (St. Louis, MO, USA). Aryl halides including 4-bromoacetophenone, 4-bromobenzaldehyde, 4-bromobenzonitrile, 4-chloroacetophenone, 4-chlorobenzaldehyde, 4-chlorobenzonitrile, 2-bromobenzonitrile, 2-chlorobenzonitrile, 2-bromopyrimidine, and 1-(5-bromothiophen-3-yl) ethenone were purchased from Sigma-Aldrich (St. Louis, MO, USA). All of the above-mentioned chemicals were used as received without further purification.

Characterization: ^{1}H NMR spectra were recorded with a Bruker 500 MHz spectrometer. UV−Vis spectra were recorded on an Agilent Cary-5 spectrophotometer and Thermo Scientific Genesys 180 UV-Visible spectrophotometer. Steady-state fluorescence spectra were measured on a HITACHI F-7000 spectrometer. The morphology of the pair of PdTPBP/PDI was characterized by using a JEOL JEM-200CX transmission electron microscope (TEM) operated at 80 kV. The sample for TEM measurement was prepared by dropping the solution onto a carbon-coated copper grid following negative staining with 5.0% (w/v) sodium phosphotungstic acid. All samples in the Stern-Volmer quenching experiments were deaerated with argon for ca. 15 min before measurement and the gas flow was maintained during the measurements. Cyclic voltammetry experiments were carried out with an electrochem luminescence analyzer instrument (LanLiKe Tianjin Chemistry and Electron High Technology Co. Ltd.) using a single-chamber electrolyzer containing a glassy carbon working electrode, a platinum wire counter electrode, and a silver-silver nitrate electrode reference electrode. For the TTA-UC spectra measurement, the diode pumped solid-state laser (653 nm, continues wave, CW, Hi-Teach company, China) was used as the excitation light source and a modified spectrofluorometer was used to record the upconversion spectra. For the photoreduction of aryl halides, red LED (625 nm), far red LED (650 nm) and blue LED (455 nm) from Mightex Company was used as the excitation light source. The photopolymerization were conducted with the 721 or 650 nm lasers coupled with fiber collimator,

which are produced by Changchun New Industries Optoelectronics Tech co., Ltd. (CNI).

Nanosecond Transient Absorption Spectra. Nanosecond transient absorption (ns-TA) spectra with excitation of 355 nm were recorded on LP920 laser flash-photolysis spectrometer (Edinburgh Instrument Ltd, UK), equipped with Nd: YAG Q-switch laser (355 nm), typical laser energy is ca. 4 mJ per pulse. The data (kinetic decay trace and spectrum) were obtained with the L900 software. All samples were deaerated with N_2 for ca. 15 min before measurement.

For ns-TA spectra with the excitation of 630 nm, a Nano-TA100 spectrometer from Time-tech Spectra was utilized. Probe beam was generated by a nanosecond Nd:YAG laser (Disco UV, Leukos, 420 nm-2400 nm). Pump beam was generated by a femtosecond laser (800 nm, 1 kHz) with TOPAS from Coherent. TOPAS was set up to generate 630 nm excitation pulse. The liquid samples (1 mm quartz cell) were settled on the platform at the intersection of the probe beam and the excitation pulse. All the samples are optically dilute at the laser excitation wavelength. The dissolved oxygen in solvent we used was removed through frozen-pump-thaw deoxygen method, and then the solvent was transferred into glove box for the preparation of solution sample. All the spectra were measured at room temperature if no further notification.

The Stern-Volmer quenching experiment[1]: The photoluminescence change of photosensitizer PdTPBP under different situation (only PdTPBP, PdTPBP with electron donor TEA, PdTPBP with model substrate 4-bromoacetophenone) were recorded upon the successive addition of photocatalyst PDI. The initial solutions were degassed for at least 15 min with argon in DMF. The k_{sv} constants were calculated with equation (1), where I_0 and I_t stand for the initial photoluminescence intensity of PdTPBP and the photoluminescence intensity of PdTPBP in the presence of PDI. Q is the concentration of PDI. Bimolecular quenching constants (k_q) were calculated by equation (2). The τ_T is the phosphorescence lifetime of photosensitizer in argon (147.5 μs[2], in toluene).

$$\frac{I_0}{I_t} = 1 + k_{sv}\,Q \quad \text{(1)}$$

$$k_{sv} = k_q \times \tau_T \quad \text{(2)}$$

The triplet-triplet energy transfer efficiency (Φ_{TTET})[3]: The PdTPBP-to-PDI triplet-triplet energy transfer quantum yield in DMF was calculated from the measurements of the PdTPBP

phosphorescence in the presence of PDI (*I*) and in the absence of PDI (I_0) using the following equation (3) that has been used in the literature.

$$\Phi_{(TTET)} = 1 - I / I_0 \quad (3)$$

The Gibbs free energy of photoinduced electron transfer (PET) (ΔG_{PET})[4]: The Gibbs free energy of photoinduced electron transfer (PET) (ΔG_{PET}) from TEA to $^3PDI^*$ was calculated according to Rehm-Weller analysis (4). F is the Faraday constant (23.061 kcal V^{-1} mol^{-1}).[5] E_{red}^* is the excited state reduction potential of PDI. Note that PDI^* refers to T_1 state, with the corresponding $E_{0,0}$ value of 1.21 eV.[6] The reduction potential of PDI was detected to be -0.44 V (vs SCE, see vide infra), thus the excited reduction potential of PDI is + 0.76 V (vs SCE)[5]. E_{ox} = + 1.0 V (vs SCE, MeCN) for triethylamine[7].

$$\Delta G_{PET} = -F\,(E_{red}^*\,(PDI^*/PDI^{\cdot -}) - E_{OX}(TEA^{\cdot +}/TEA)) \quad (4)$$

$$E_{red}^*\,(PDI^*/PDI^{\cdot -}) = E_{red}\,(PDI/PDI^{\cdot -}) + E_{0,0} \quad (5)$$

Radical anion generation measurement[8]: Photosensitizer PdTPBP, photocatalyst PDI and electron donor TEA were mixed in DMF within a quartz cuvette and then degassed for at least 15 min with argon. Then, the cuvette was excited with the blue LED (455 nm), red LED (625 nm), far-red LED (650 nm) and devoted to UV-vis absorption measurement after different irradiation time.

The TTA-UC spectra measurement[9]: A continuous diode-pumped solid-state laser (653 nm) was used as the excitation source for the upconversion measurement. PdTPBP and PDI were mixed in different solutions and then degassed with argon for 15 min. The argon gas flow was maintained during the measurement. The treated solution was then excited with the laser, and the corrected upconversion spectrum was recorded with a HORIBA spectrofluorometer.

The measurement and calculation of upconversion efficiency (Φ_{UC}): The Φ_{UC} was calculated by an established method[10,11]. Methylene blue (MB) in methanol with fluorescence quantum yield (Φ_{std} = 3%) was used as the reference[12]. The upconversion efficiency were calculated with the equation (6), where Φ_{UC} stands for the upconversion efficiency of the testing sample. A_{unk} and A_{std}

stand for absorbance of the TTA-UC sample and the reference compound, respectively. I_{unk} and I_{std} stand for the integrated upconversion luminescence intensity of the TTA-UC sample and the fluorescence intensity of the reference compounds, respectively. η_{unk} and η_{std} stand for the refractive index of solvents of the TTA-UC sample and the reference compound. The equation is multiplied by a factor of 2 to make the maximum quantum yield of two unified emitters.

$$\Phi_{UC} = 2 \times \Phi_{std} \times \frac{A_{std}}{A_{unk}} \times \frac{I_{unk}}{I_{std}} \times \left(\frac{\eta_{unk}}{\eta_{std}}\right)^2 \quad \text{.. (6)}$$

Photoreduction of 4-bromoacetophenone with PDI: Photocatalyst PDI (2.0 mg, 1.25 mM), electron donor TEA (57 µL, 200 mM) and 4-bromoacetophenone (10 mg, 25 mM) were mixed in anhydrous DMF (2 mL) and then degassed for at least 15 min with argon. Then, the solution was excited by a deep blue LED (455 nm, 100 mW/cm^2) at 40 ºC. After the photoreduction, the raw product was diluted with ether (3 mL), and then 3 mL 1 M HCl was added. This mixture was stirred for 3 minutes, and the upper organic layer was collected. 3 mL saturated sodium chloride solution was added to the organic layer to wash the HCl. The upper ether layer was collected. The product yields were measured via gas chromatography with internal standard of benzonitrile (Supplementary Figure 29-33). The GC spectrum of the standard sample consisting of the equivalent amount of benzonitrile, acetophenone and 4-bromoacetophenone, is provided as Supplementary Figure 35.

Photoreduction of 4-bromoacetophenone with PdTPBP/PDI pair: Photosensitizer PdTPBP (50 µM), photocatalyst PDI (2.0 mg, 1.25 mM), electron donor TEA (57 µL, 200 mM) and 4-bromoacetophenone (10 mg, 25 mM) were mixed in anhydrous DMF (2 mL) and then degassed for at least 15 min with argon. Then the solution was excited with a red-light LED (625 nm) or far red-light LED (655 nm) (100 mW/cm^2) at 40 ºC. After completed reaction, the raw product was diluted with ether (3 mL), and then 3 mL 1 M HCl was added. This mixture was stirred for 3 minutes, and the upper organic layer was collected. 3 mL of saturated sodium chloride (NaCl) solution was added to the organic layer to wash the HCl. The upper ether layer was collected. The product yields were measured via gas chromatography with internal standard of benzonitrile (Supplementary Figure 25-28 and 34).

Photoreduction of other aryl halides with PdTPBP/PDI pair: Photosensitizer PdTPBP (50 μM), photocatalyst PDI (2.0 mg, 1.25 mM), electron donor TEA (57 μL, 200 mM) and aryl halides (10 mg, 25 mM) were mixed in anhydrous DMF (2 mL) and then degassed for at least 15 min with argon. Then the solution was excited with a far red-light LED (650 nm, 100 mW/cm^2) at 40 ºC. After 16 hours reaction, the raw product was diluted with ether (5 mL), and then 5 mL 1 M HCl was added. This mixture was stirred for 3 minutes, and the upper organic layer was collected. Add 5 mL of saturated sodium chloride (NaCl) solution to the organic layer to wash the HCl. The upper ether layer was collected, and removed the ether. The raw product was purified with small silica column.

Long-wavelength light-driven photopolymerization to produce gel via TS-conPET process: Firstly, the monomer of MMA and the crosslinker of ethylene glycol dimethacrylate were purified through a small column of aluminum oxide to remove the stabilizer. Then, photosensitizer PtTNP or BDP (0.034 μmol), photocatalyst PDI (0.7 mg, 1 μmol), electron donor TEA (69 μL, 0.5 mmol), MMA (1.07 mL, 10 mmol), EGD (188 μL, 1 mmol), EBrP (65 μL, 0.5 mmol) and anhydrous DMF (0.5 mL) were mixed in a tube within the glove box and then sealed to be kept in a deoxygenated atmosphere. Then the solution was excited with NIR light (for PtTNP, 721 nm) or far-red light (for BDP, 650 nm) at room temperature.

Supplementary Tables

Supplementary Table 1. The photophysical properties of photosensitizers and photocatalyst used in this work.[a]

Compound	λ_{abs} (nm)[b]	ε (cm^{-1} M^{-1})[c]	λ_{em} (nm)[d]	Φ_L (%) [e]	τ_T (μs)[f]	T_1 (eV)[g]
PdTPBP	628	1.1×10^5	790	16.7[h]	147.5[h]	1.56
PDI	491/527	0.90×10^5	545/581	100	—	1.20[i]
PtTNP[j]	689	1.2×10^5	872	15	12.7	1.43
BDP[k]	616	1.76×10^5	635	12.7	—	1.44

[a] In DMF, 10 μM; [b] maximum absorption wavelength; [c] molar absorption coefficient; [d] the fluorescence peak wavelength; [e] luminescence quantum yield; [f] triplet excited state lifetime; [g] triplet excited energy level; [h] reference [2]; [i] reference [6]; [j] reference [13] ; [k] reference [14].

Supplementary Table 2. Kinetic data acquired from the biexponential analysis of the triplet excited lifetime of PdTPBP dynamics as a function of PDI concentration using the ESA decay trace of PdTPBP at 535 nm in DMF. For all the samples, the concentrations of PdTPBP are all 10 μM.

535 nm	τ_{T-1}(μs)	τ_{T-2} (μs)
PdTPBP	161.7	–
PdTPBP/PDI = 1/1	37.9	339.3
PdTPBP/PDI = 1/2	21.8	526.8
PdTPBP/PDI = 1/3	18.6	522.3

Supplementary Table 3. Kinetic data acquired from the exponential analysis of the triplet excited lifetime of PdTPBP dynamics as a function of PDI concentration using the GSB decay trace of PdTPBP at 640 nm in DMF. For all the samples, the concentrations of PdTPBP are all 10 μM.

640 nm	τ_T (μs)
PdTPBP	161.7
PdTPBP/PDI = 1/1	36.2
PdTPBP/PDI = 1/2	22.2
PdTPBP/PDI = 1/3	18.3

Supplementary Table 4. Stern−Volmer quenching constant (k_{sv}), bimolecular quenching constant (k_q), and TTET quantum efficiency (Φ_{TTET}) of PdTPBP and PDI under different conditions.

	toluene	DMF	DMF + TEA	DMF + 4-bromoacetophenone
k_{sv} (M^{-1})	8.1×10^5	7.2×10^5	8.3×10^5	7.09×10^5
k_q (M^{-1} s^{-1})	5.49×10^9	4.88×10^9	5.63×10^9	4.81×10^9
Φ_{TTET} (%) [a]	84.3	76.0	82.4	75.1

[a] PdTPBP (10 μM), PDI (8.0 μM).

Supplementary Table 5. Control experiments to verify the key parameters for the photoreduction of 4-bromoacetophenone via triplet sensitization pathway.

Entry	PdTPBP	PDI	Light	TEA	Yield (%)
1	+	+	−	+	trace
2	+	+	+	−	trace
3	−	+	+	+	trace
4	+	−	+	+	trace

Supplementary Table 6. Excitation wavelength of typical reported photocatalysts to conduct conPET process.

Photocatalyst	Wavelength(nm)	Reference
PDI	455/530	*Science* **346**, 725 (2014)
Rh-6G	455	*Angew. Chem. Int. Ed.* **55,** 7676-7679 (2016)
$[Ru(bpy)_3]^{2+}$/ pyrene-1-carboxylate	532	*Chem Sci.* **7**, 3862–3868 (2016)
$[Ru(bpy)_3]^{2+}$	520	*Angew. Chem. Int. Ed.* **57**, 1078–1081 (2018)
BPI	405	*J. Am. Chem. Soc.* **142,** 13573-13581 (2020)
Mes-$Acr^{+}BF_4^{-}$	390	*Nature* **580,** 76–80 (2020)
$Ir(dtbby)(ppy)_2PF_6$	455	*Nat. Catal.* ***3,*** 40–47 (2020)
3CzEPAIPN	456/525	*J. Am. Chem. Soc.* **143**, 13266-13273, (2021)
$[Cu(dap)_2]^{+}$/DCA	623/635	*JACS Au*. **2**, 1488–1503 (2022)

Supplementary Figures

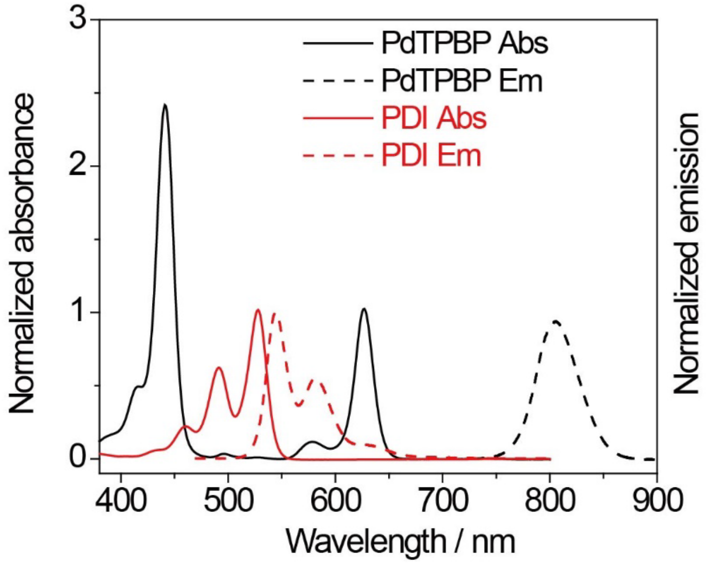

Supplementary Figure 1. Normalized absorption and emission spectra of PdTPBP and PDI in DMF. PDI (λ_{ex} = 450 nm), PdTPBP (λ_{ex} = 630 nm).

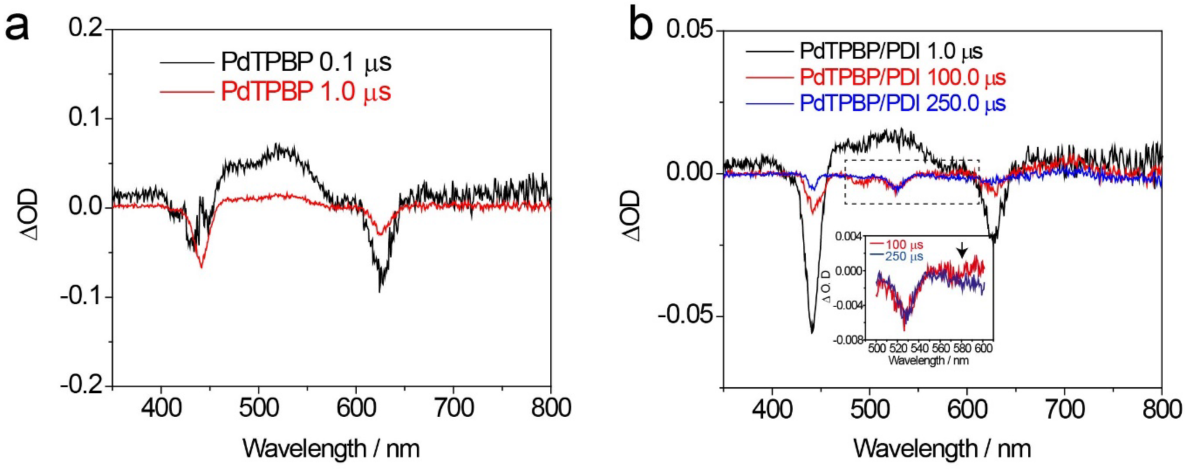

Supplementary Figure 2. **Triplet sensitization between PdTPBP and PDI via transient absorption analysis.** Nanosecond transient absorption spectra of (**a**) PdTPBP at different delay times (0.1 and 1.0 μs) and (**b**) PdTPBP/PDI pair (1:1 molar ratio) at different delay times (1.0, 100 and 250 μs). λ_{ex} = 355 nm

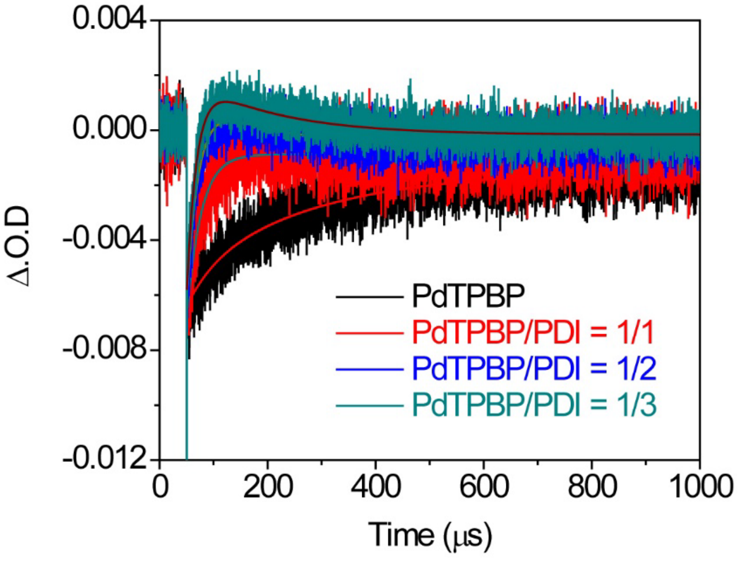

Supplementary Figure 3. Decay traces of the ground-state bleaching signal of PdTPBP at 640 nm with different molar ratios of PdTPBP/PDI. c (PdTPBP) = 10 μM, in deaerated DMF.

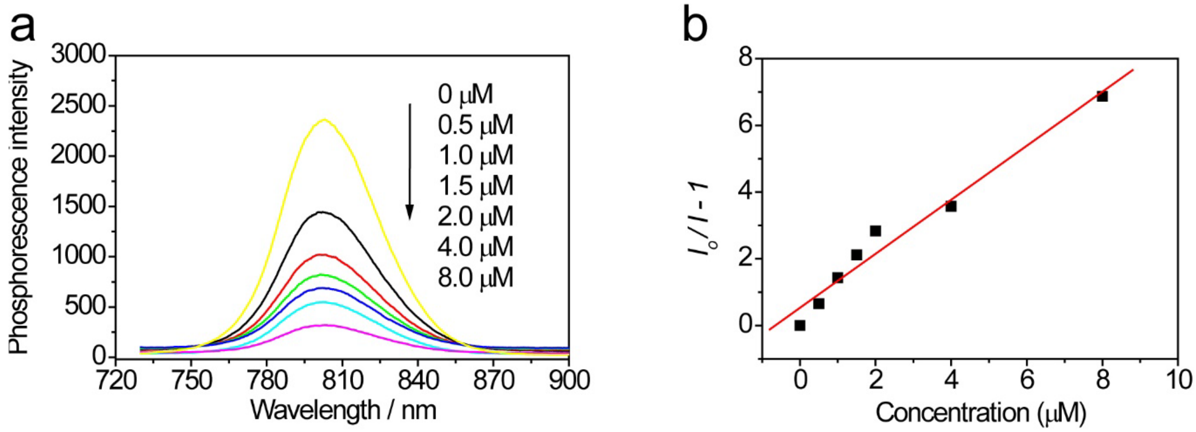

Supplementary Figure 4. Quenching of PdTPBP phosphorescence by PDI. (**a**) Phosphorescence intensity change of PdTPBP via titration of PDI in toluene; (**b**) Stern−Volmer plots of PdTPBP in the presence of PDI in toluene. C (PdTPBP) = 10 μM, λ_{ex} = 630 nm.

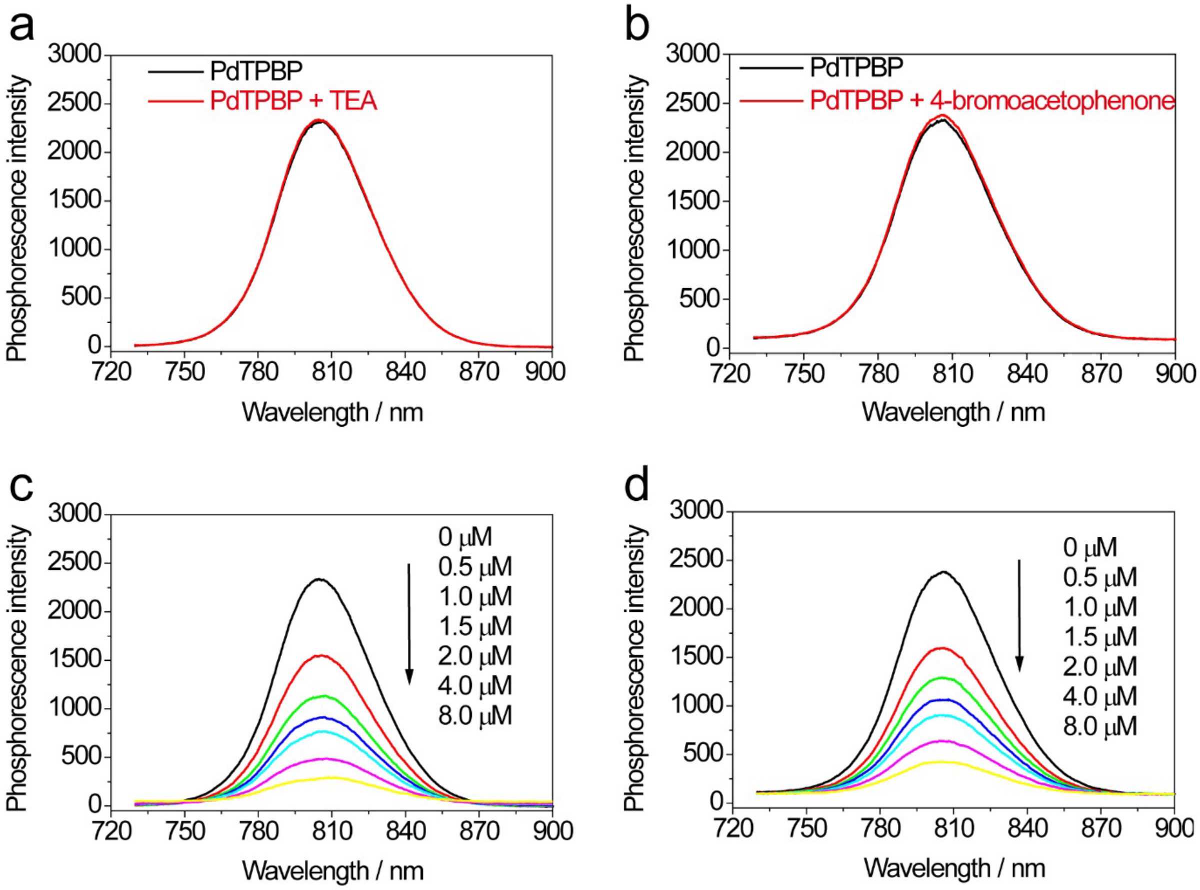

Supplementary Figure 5. Quenching of PdTPBP phosphorescence by PDI with additives. (**a**) Phosphorescence emission spectra of PdTPBP (10 μM) with or without TEA (100 eq) in DMF; (**b**) Phosphorescence emission spectra of PdTPBP (10 μM) with or without 4-bromoacetophenone (100 eq) in DMF; (**c**) Phosphorescence intensity change of PdTPBP (10 μM) via titration of PDI in DMF in the presence of TEA (100 eq); (**d**) Phosphorescence intensity change of PdTPBP (10 μM) via titration of PDI in DMF in the presence of 4-bromoacetophenone (100 eq). λ_{ex} = 630 nm.

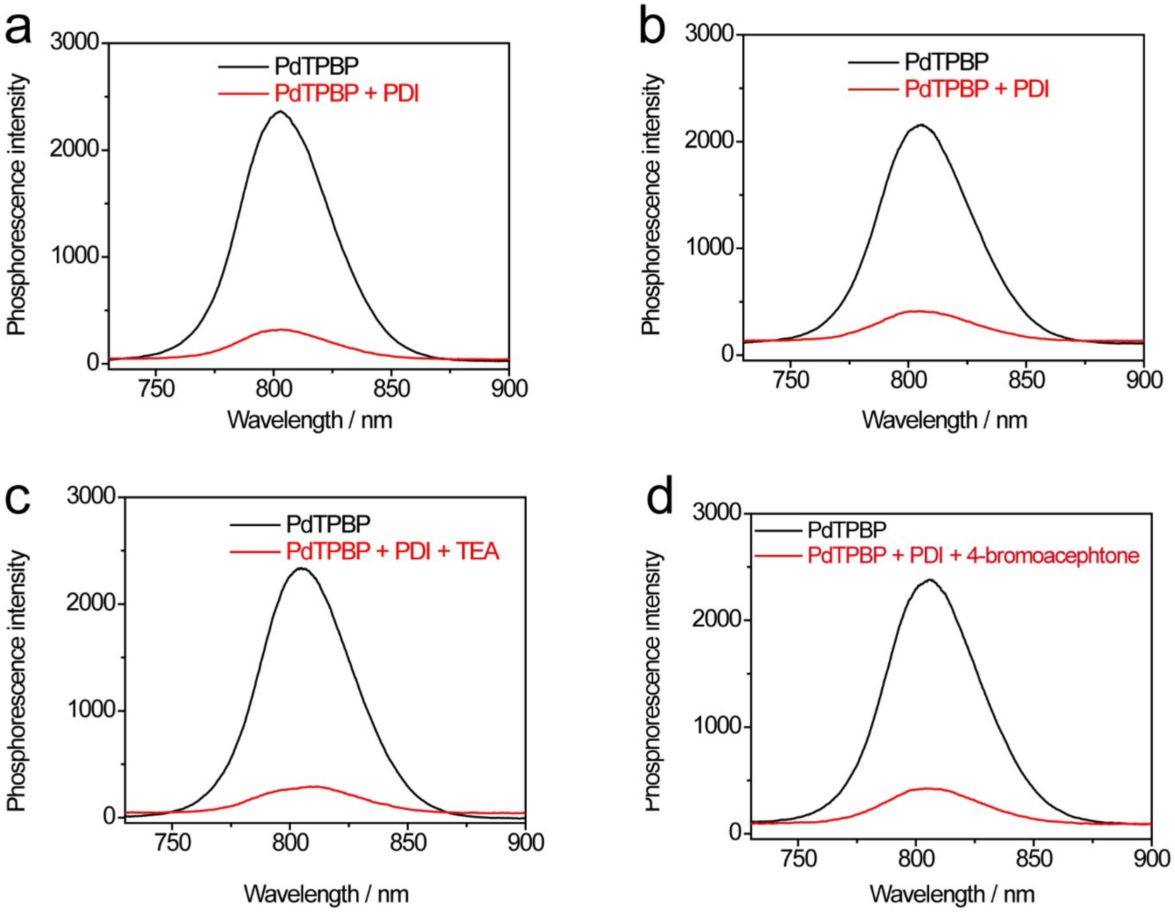

Supplementary Figure 6. Solvent influence on the quenching of PdTPBP phosphorescence by PDI with additives. Phosphorescence emission spectra of PdTPBP (10 μM) without PDI and in the presence of PDI (8.0 μM) under various conditions, λ_{ex} =630 nm. (**a**) in toluene, (**b-d**) in DMF.

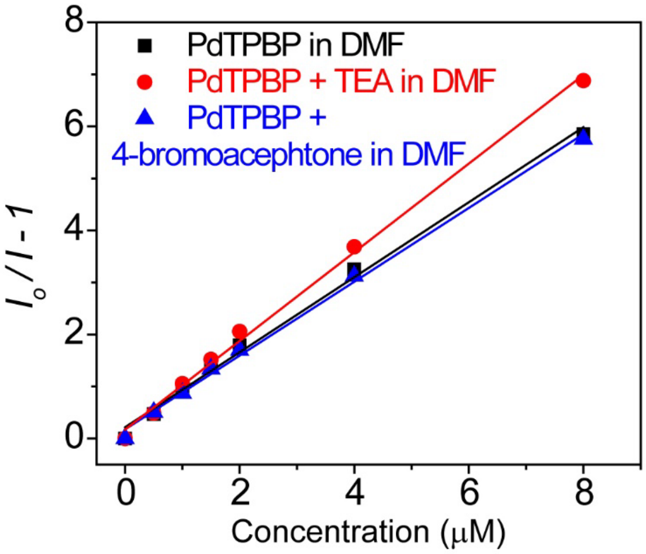

Supplementary Figure 7. Stern−Volmer quenching plots of PdTPBP with PDI under various conditions (only PdTPBP, PdTPBP with 100 eq TEA, and PdTPBP with 100 eq 4-bromoacetophenone). C (PdTPBP) = 10 μM, λ_{ex} = 630 nm, in DMF.

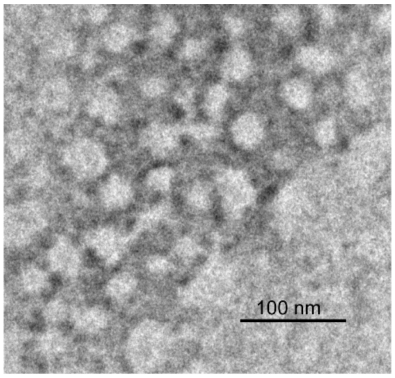

Supplementary Figure 8. TEM image of the PdTPBP/PDI pair. Dark region is the sodium phosphotungstate straining and the white region is the nano-aggregation of PdTPBP/PDI.

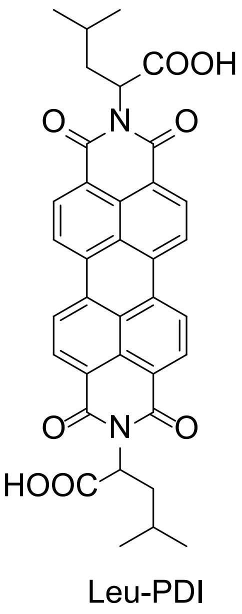

Supplementary Figure 9. Molecular structure of Leu-PDI.

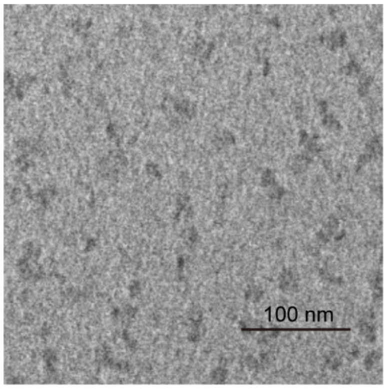

Supplementary Figure 10. TEM image of the PdTPBP/Leu-PDI pair. Dark region is the phosphotungstic acid staining.

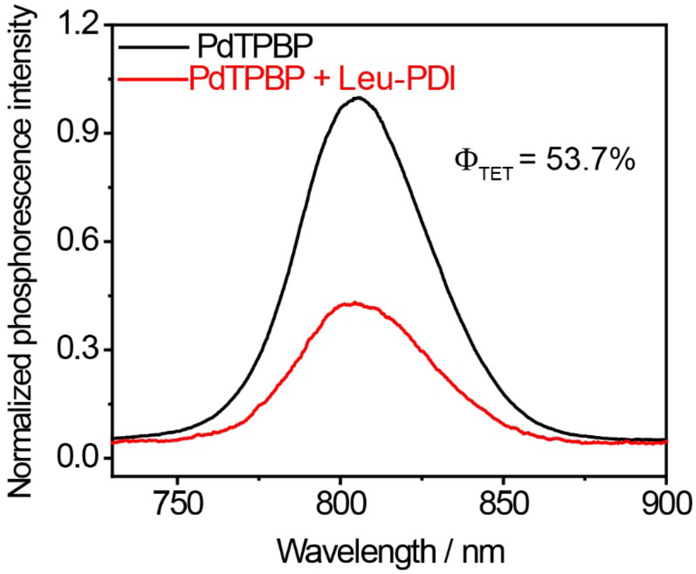

Supplementary Figure 11. Phosphorescence emission spectra of PdTPBP (10 μM) without Leu-PDI and in the presence of Leu-PDI (8.0 μM), λ_{ex} =630 nm, in DMF.

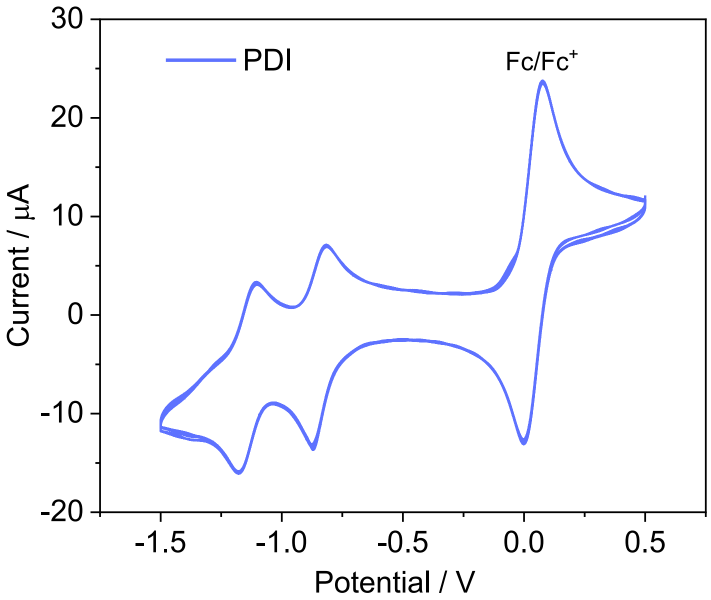

Supplementary Figure 12. Cyclic voltammogram of PDI. Ferrocene (Fc) was used as an internal reference. Condition: in deaerated DMF, 0.10 M Bu_4NPF_6 as the supporting electrolyte and $Ag/AgNO_3$ as a reference electrode. Scan rates: 100 mV/s, 25 °C. E_{red} (PDI/ $PDI^{\bullet -}$) = -0.89 V (vs Fc/Fc^+) = -0.44 V (vs SCE), which is close to the reported value of -0.43 V (vs SCE).[15]

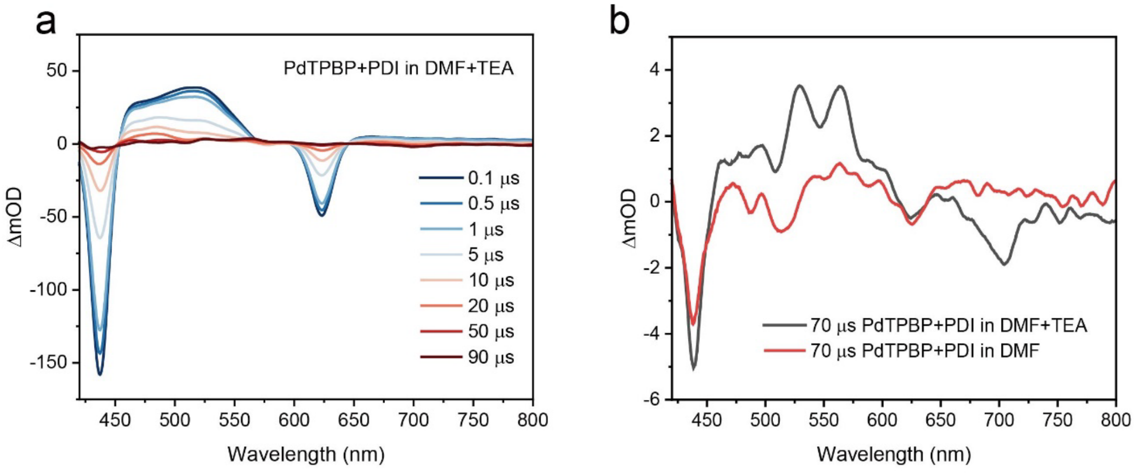

Supplementary Figure 13. **Triplet sensitization between PdTPBP and PDI under various conditions via transient absorption analysis.** Nanosecond transient absorption spectra for (**a**) the mixture of PdTPBP/PDI and TEA in DMF at different delay times (0.1~90 μs) and (**b**) the decay traces of PdTPBP/PDI in DMF with or without TEA at delay time of 70 μs. λ_{ex} = 630 nm, *c* (PdTPBP) = *c* (PDI) = 10 μM.

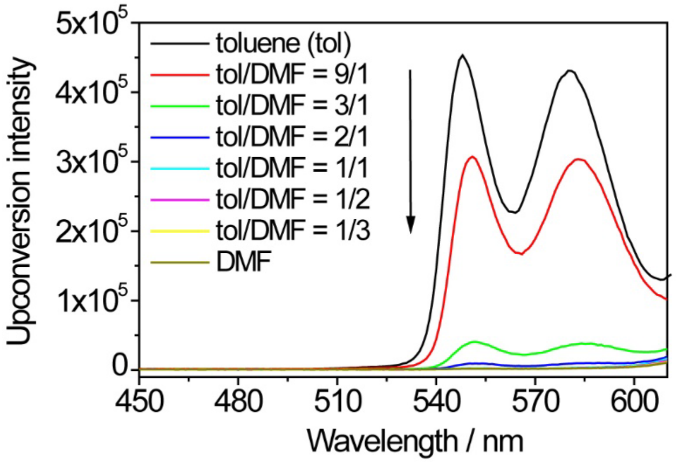

Supplementary Figure 14. TTA-UC spectra of PdTPBP (10 μM)/PDI (50 μM) in solutions with different ratios of toluene/DMF. Excitation light is 650 nm with power intensity of 100 mW/cm^2.

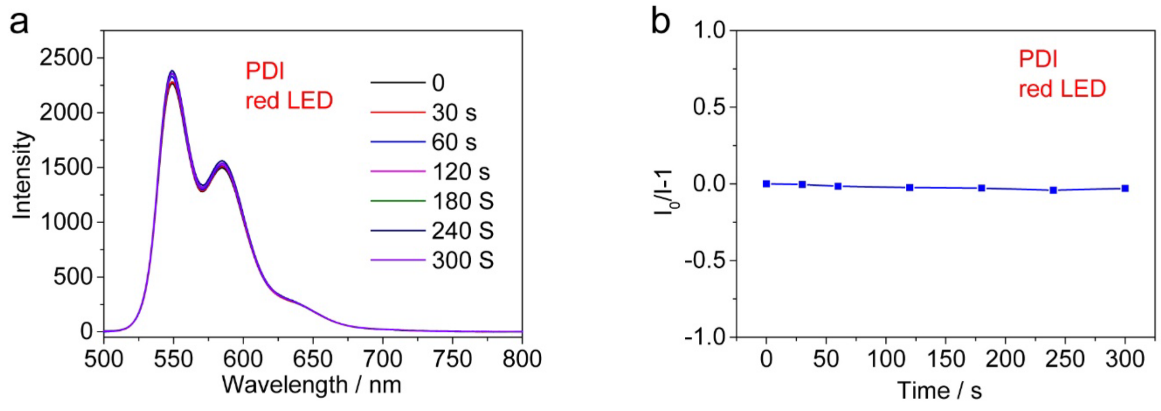

Supplementary Figure 15. **Fluorescence quenching analysis of PDI by TEA.** (**a**) The fluorescence quenching spectra and (**b**) the related Stern-Volmer plot of PDI in the presence of TEA under 625 nm LED (20 mW/cm^2) illumination, in DMF.

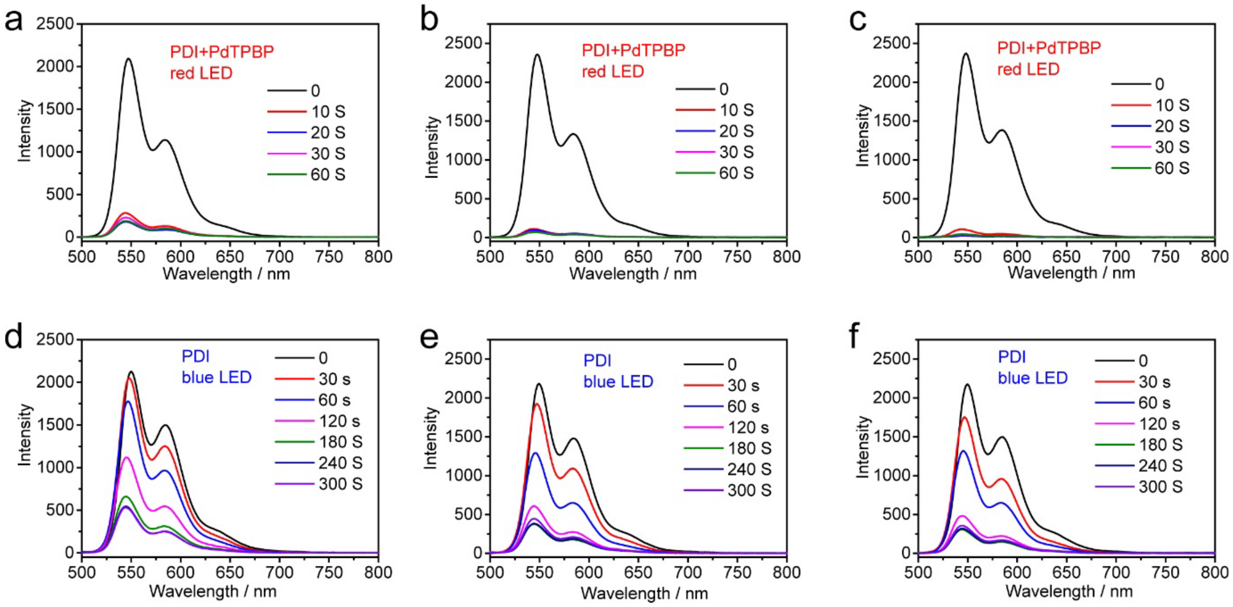

Supplementary Figure 16. **The quenching of PDI fluorescence under light irradiation with different power intensity.** (**a-c**) The pair of PdTPBP/PDI in the presence of TEA: **a**, 40 mW/cm^2; **b**, 60 mW/cm^2; **c**, 80 mW/cm^2. λ_{ex} = 625 nm LED, OD (625 nm) = 0.9 for PdTPBP. (**d-f**) The PDI alone in the presence of TEA: **d**, 40 mW/cm^2; **e**, 60 mW/cm^2; **f**, 80 mW/cm^2. λ_{ex} = 455 nm LED, OD (455 nm) = 0.9 for PDI. In DMF, TEA: 30 μL.

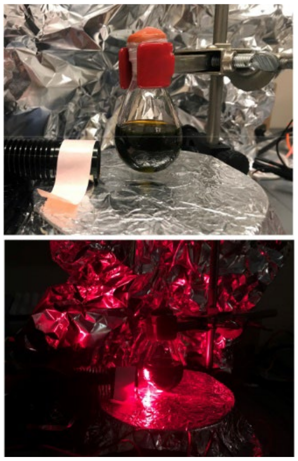

Supplementary Figure 17. The photoreduction setup for 20 mL reaction: the LED beam size is 2.5 cm, diameter of reactor is 3.9 cm, and the magnetic stirring rate is 500 rpm.

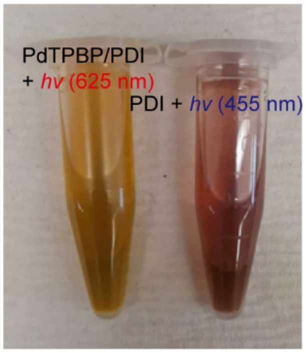

Supplementary Figure 18. The picture of PdTPBP/PDI and PDI after 16 hours of LED irradiation in the presence of TEA in DMF.

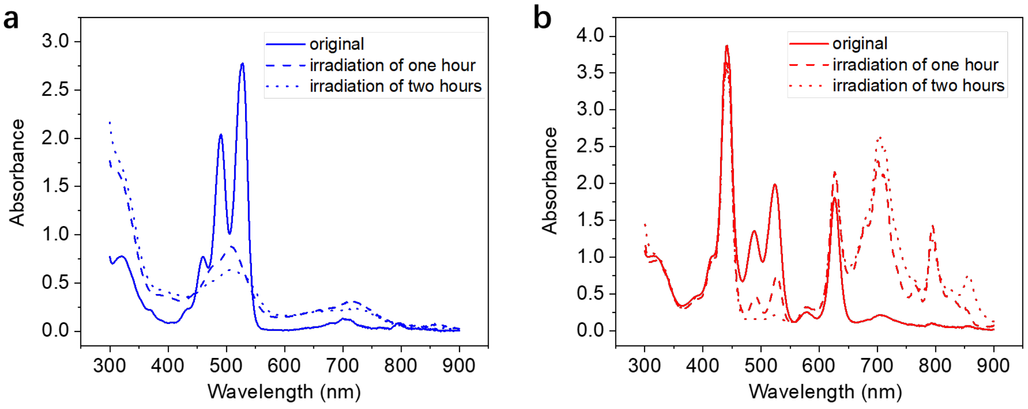

Supplementary Figure 19. UV-vis absorption change of PDI in the presence of TEA under different illumination. UV-vis absorption change of (**a**) the mixed solution of PDI and TEA in deoxygenated DMF upon 455 nm LED irradiation (100 mW/cm^2) or (**b**) the mixed solution of PDI, PdTPBP and TEA in deoxygenated DMF upon 650 nm LED irradiation (100 mW/cm^2).

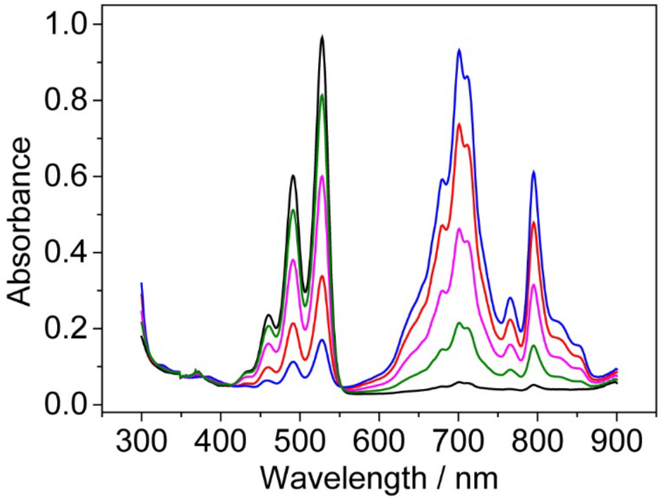

Supplementary Figure 20. Blue light (455 nm) driven PDI$^{\bullet -}$ generation in the presence of triethylamine (TEA). The absorption of 600-900 nm is the featured peak of PDI$^{\bullet -}$, which are consistent with literature[8].

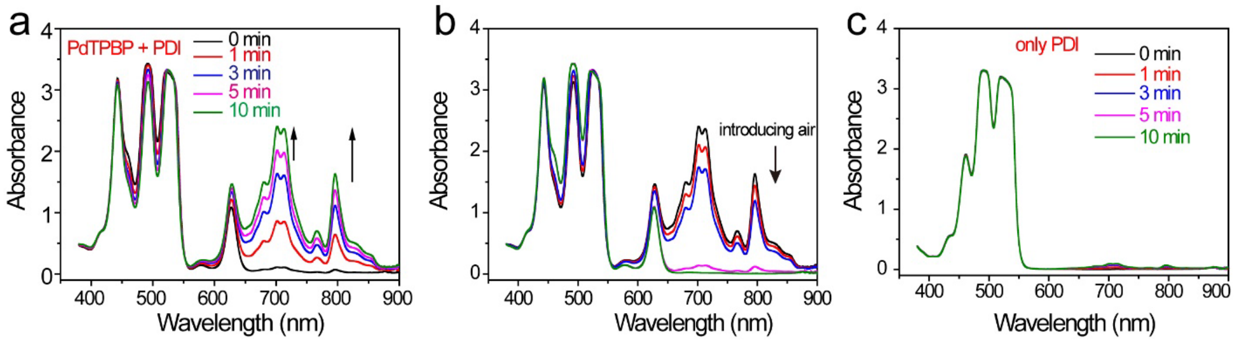

Supplementary Figure 21. **UV-vis absorption spectra of different solutions with TEA in DMF**: (**a**) PDI and PdTPBP mixture in Ar, 650 nm LED illumination (20 mW/cm^2); (**b**) introducing air into the irradiated PDI and PdTPBP mixture; (**c**) PDI alone under 650 nm light LED illumination. c (PdTPBP) = 10 μM, c (PDI) = 25 μM, TEA 30 μL.

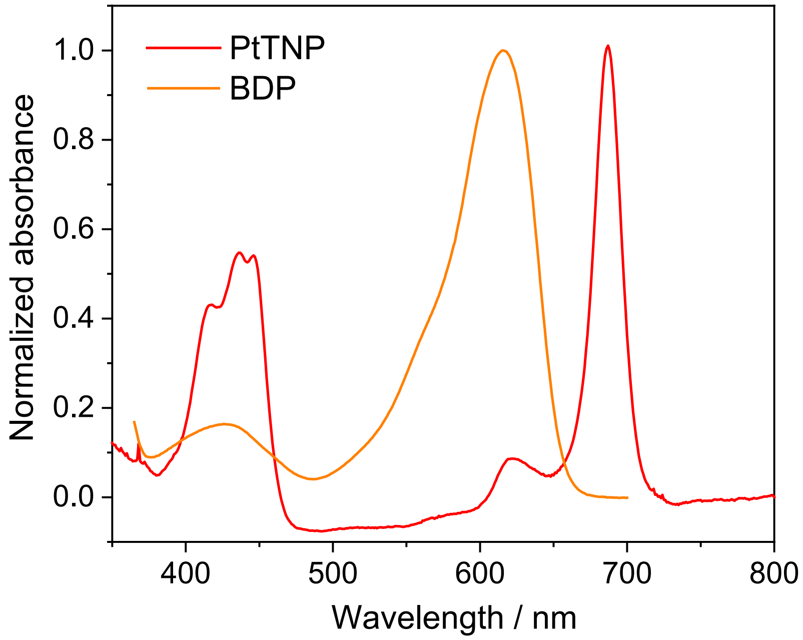

Supplementary Figure 22. Normalized UV-vis absorption of photosensitizers BDP and PtTNP.

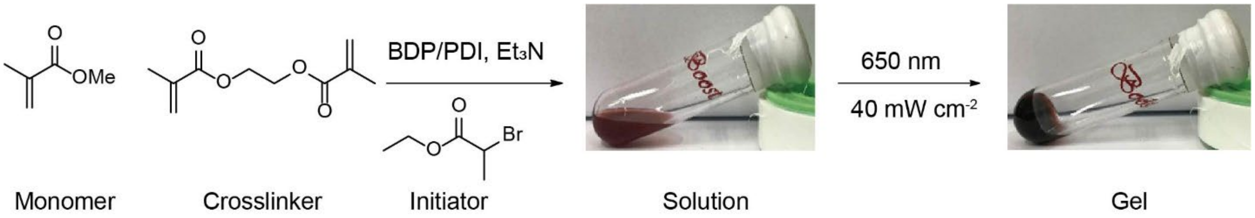

Supplementary Figure 23. Red light-driven photopolymerization to produce gel with BDP/PDI pair via TS-conPET process.

Entry	Variations from standard condition	Before reaction	After reaction
1	Without BDP<br>450 nm irradiation	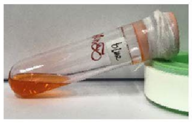	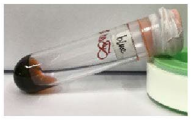
2	Without BDP	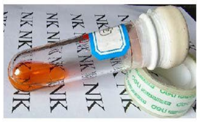	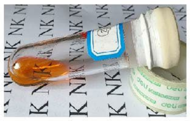
3	Without BDP, in dark	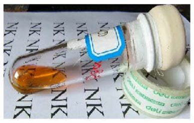	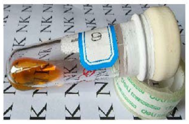
4	Without BDP/PDI<br>450 nm irradiation	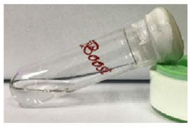	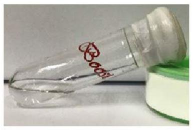
5	Without PDI	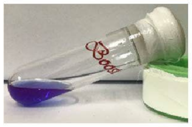	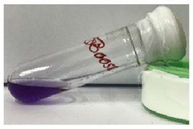
6	BDP replaced by PtTNP<br>721 nm irradiation	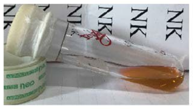	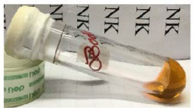
7	BDP replaced by PtTNP<br>without PDI, 721 nm irradiation	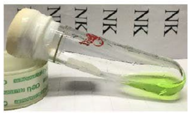	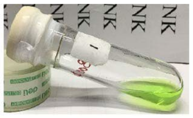

Supplementary Figure 24. Control experiments and extensions with the reaction setup in Supplementary Figure 23 as the standard condition.

Gas chromatography data for the photoreductions

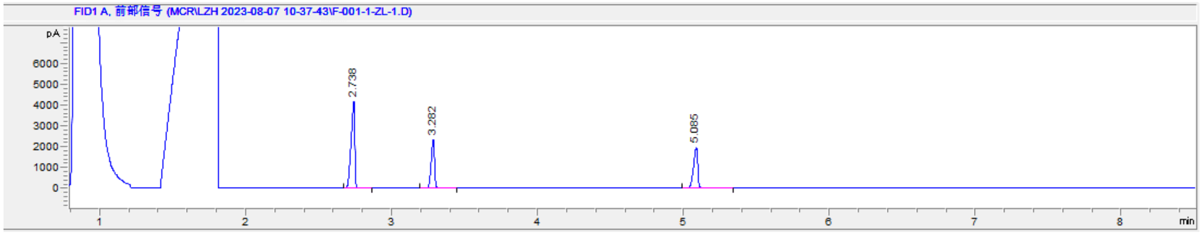

Supplementary Figure 25. Gas chromatography (GC) spectrum for the photoreduction of 4-bromoacetophone with PdTPBP/PDI pair in 2 mL DMF after two hours of 625 nm LED irradiation.

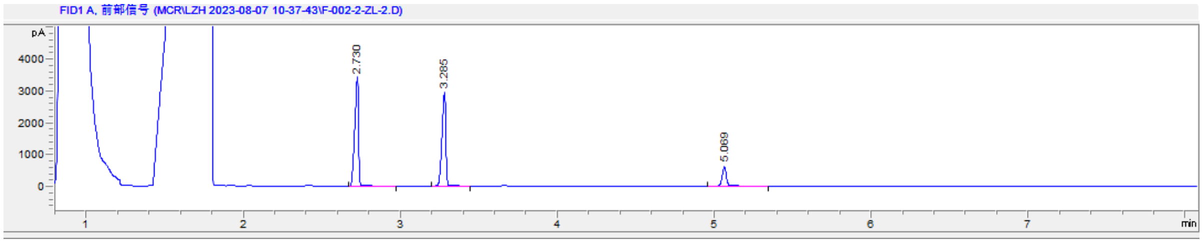

Supplementary Figure 26. GC spectrum for the photoreduction of 4-bromoacetophone with PdTPBP/PDI pair in 2 mL DMF after four hours of 625 nm LED irradiation.

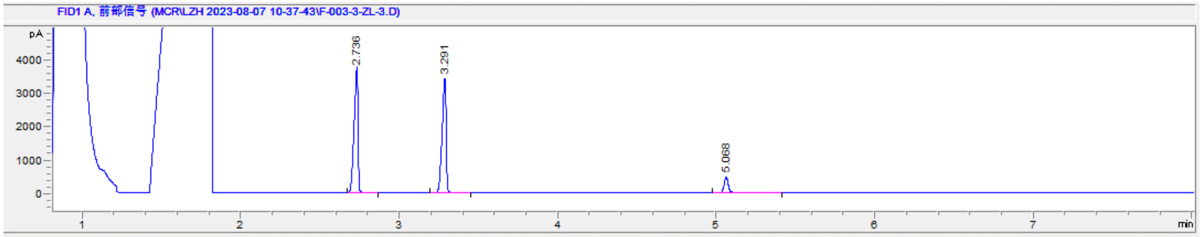

Supplementary Figure 27. GC spectrum for the photoreduction of 4-bromoacetophone with PdTPBP/PDI pair in 2 mL DMF after six hours of 625 nm LED irradiation.

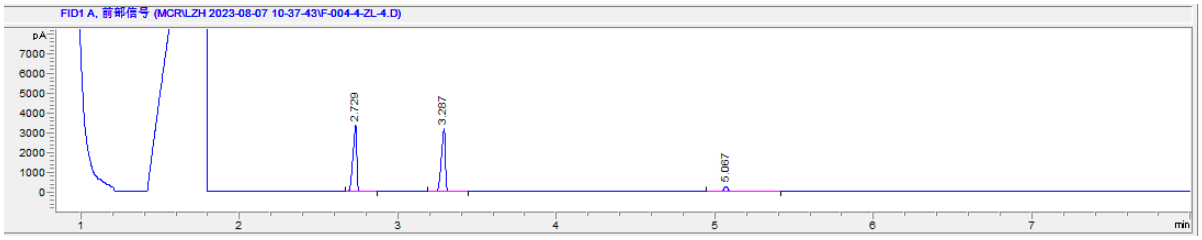

Supplementary Figure 28. GC spectrum for the photoreduction of 4-bromoacetophone with PdTPBP/PDI pair in 2 mL DMF after eight hours of 625 nm LED irradiation.

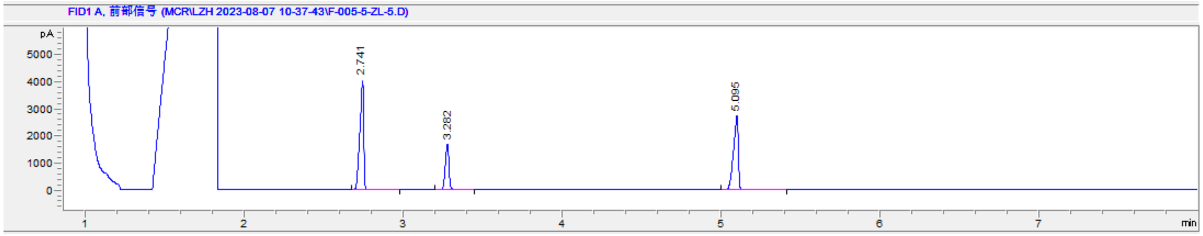

Supplementary Figure 29. GC spectrum for the photoreduction of 4-bromoacetophone with PDI in 2 mL DMF after two hours of 455 nm LED irradiation.

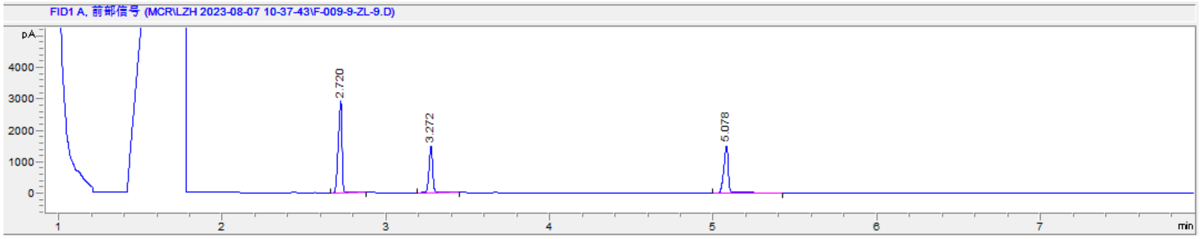

Supplementary Figure 30. GC spectrum for the photoreduction of 4-bromoacetophone with PDI in 2 mL DMF after four hours of 455 nm LED irradiation.

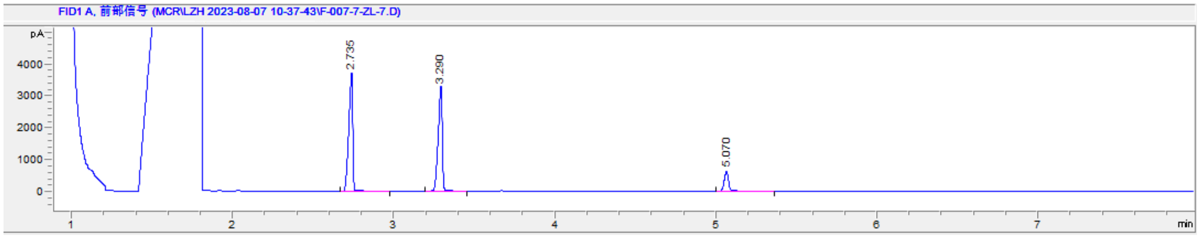

Supplementary Figure 31. GC spectrum for the photoreduction of 4-bromoacetophone with PDI in 2 mL DMF after six hours of 455 nm LED irradiation.

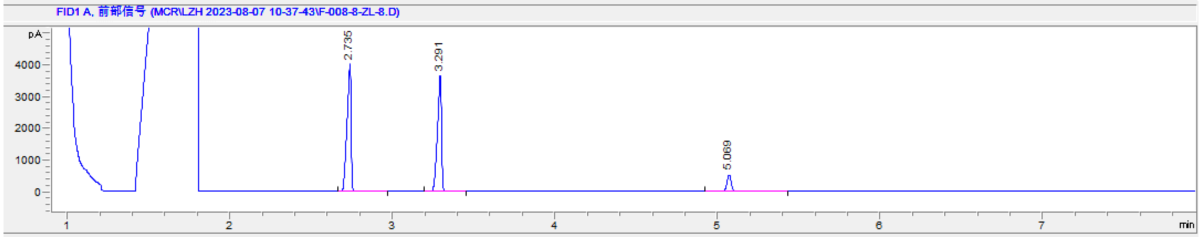

Supplementary Figure 32. GC spectrum for the photoreduction of 4-bromoacetophone with PDI in 2 mL DMF after eight hours of 455 nm LED irradiation.

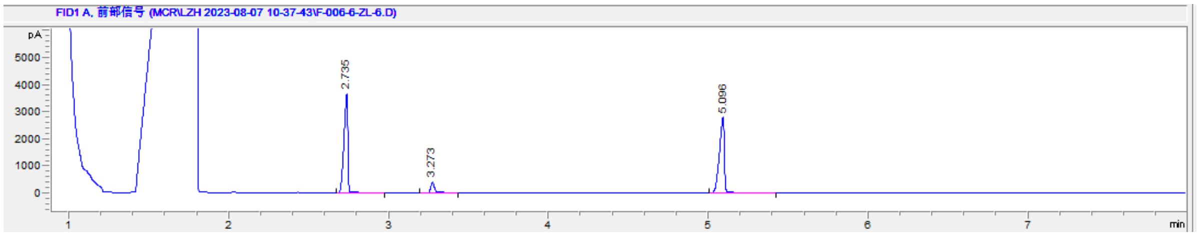

Supplementary Figure 33. GC spectrum for the photoreduction of 4-bromoacetophone with PDI in 20 mL DMF after four hours of 455 nm LED irradiation.

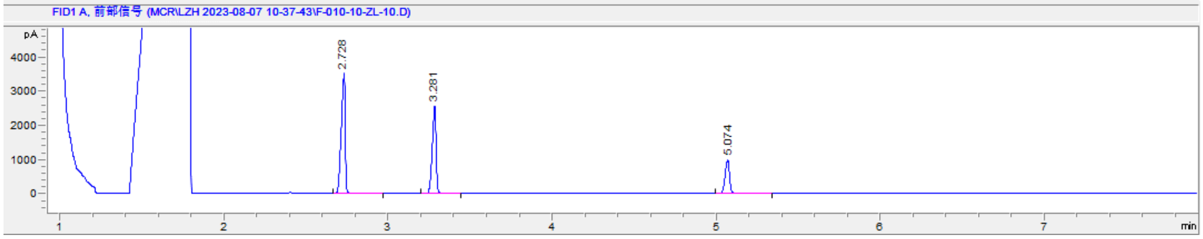

Supplementary Figure 34. GC spectrum for the photoreduction of 4-bromoacetophone with PdTPBP/PDI pair in 20 mL DMF after four hours of 625 nm LED irradiation.

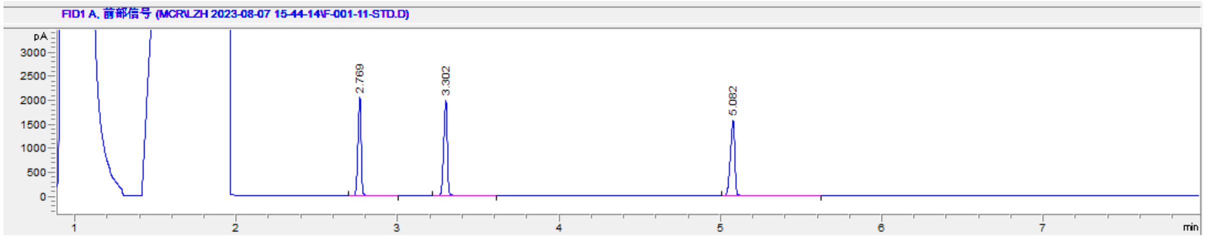

Supplementary Figure 35. GC spectrum for the standard sample consisting of the equivalent amount of benzonitrile (t = 2.769), acetophenone (t = 3.302) and 4-bromoacetophenone (t = 5.082).

Supplementary References

1 Zhao, J., Ji, S. & Guo, H. Triplet–triplet annihilation based upconversion: from triplet sensitizers and triplet acceptors to upconversion quantum yields. *RSC Adv.* **1**, 937-950, doi:10.1039/C1RA00469G (2011).

2 Cui, X., Zhao, J., Yang, P. & Sun, J. Zinc(ii) tetraphenyltetrabenzoporphyrin complex as triplet photosensitizer for triplet–triplet annihilation upconversion. *Chem. Commun.* **49**, 10221-10223, doi:10.1039/C3CC45843A (2013).

3 Mattiello, S. *et al.* Self-Assembled Dual Dye-Doped Nanosized Micelles for High-Contrast Up-Conversion Bioimaging. *Advanced Functional Materials* **26**, 8447-8454, doi:https://doi.org/10.1002/adfm.201603303 (2016).

4 Rehm, D. & Weller, A. Kinetics of Fluorescence Quenching by Electron and H-Atom Transfer. *Isr. J. Chem.* **8**, 259-271, doi:https://doi.org/10.1002/ijch.197000029 (1970).

5 Romero, N. A. & Nicewicz, D. A. Organic Photoredox Catalysis. *Chem. Rev.* **116**, 10075-10166, doi:10.1021/acs.chemrev.6b00057 (2016).

6 Singh-Rachford, T. N. *et al.* Supermolecular-Chromophore-Sensitized Near-Infrared-to-Visible Photon Upconversion. *J. Am. Chem. Soc.* **132**, 14203-14211, doi:10.1021/ja105510k (2010).

7 Prier, C. K., Rankic, D. A. & MacMillan, D. W. C. Visible Light Photoredox Catalysis with Transition Metal Complexes: Applications in Organic Synthesis. *Chem. Rev.* **113**, 5322-5363, doi:10.1021/cr300503r (2013).

8 Ghosh, I., Ghosh, T., Bardagi, J. I. & Konig, B. Reduction of aryl halides by consecutive visible light-induced electron transfer processes. *Science* **346**, 725-728, doi:10.1126/science.1258232 (2014).

9 Singh-Rachford, T. N., Haefele, A., Ziessel, R. & Castellano, F. N. Boron Dipyrromethene Chromophores: Next Generation Triplet Acceptors/Annihilators for Low Power Upconversion Schemes. *J. Am. Chem. Soc.* **130**, 16164-16165, doi:10.1021/ja807056a (2008).

10 Kerzig, C. & Wenger, O. S. Sensitized triplet-triplet annihilation upconversion in water and its application to photochemical transformations. *Chem. Sci.* **9**, 6670-6678, doi:10.1039/c8sc01829d (2018).

11 Zhou, J., Liu, Q., Feng, W., Sun, Y. & Li, F. Upconversion Luminescent Materials: Advances and Applications. *Chem. Rev.* **115**, 395-465, doi:10.1021/cr400478f (2015).

12 Liu, Q. *et al.* A General Strategy for Biocompatible, High-Effective Upconversion Nanocapsules Based on Triplet–Triplet Annihilation. *J. Am. Chem. Soc.* **135**, 5029-5037, doi:10.1021/ja3104268 (2013).

13 Huang, L. *et al.* Long wavelength single photon like driven photolysis via triplet triplet annihilation. *Nat. Commun.* **12**, 122, doi:10.1038/s41467-020-20326-6 (2021).

14 Zeng, L. *et al.* Metal-Free Far-Red Light-Driven Photolysis via Triplet Fusion to Enhance Checkpoint Blockade Immunotherapy. *Angew. Chem. Int. Ed.* **62**, e202218341, doi:https://doi.org/10.1002/anie.202218341 (2023).

15 Zeman, C. J. t., Kim, S., Zhang, F. & Schanze, K. S. Direct observation of the reduction of aryl halides by a photoexcited perylene diimide radical anion. *J. Am. Chem. Soc.* **142**, 2204-2207, doi:10.1021/jacs.9b13027 (2020).
